# Psychiatric Symptoms, Treatment Uptake, and Barriers to Mental Health Care Among US Adults With Post–COVID-19 Condition

**DOI:** 10.1001/jamanetworkopen.2024.8481

**Published:** 2024-04-25

**Authors:** Hiten Naik, Karen C. Tran, John A. Staples, Roy H. Perlis, Adeera Levin

**Affiliations:** 1 Department of Medicine, The University of British Columbia, Vancouver, British Columbia, Canada; 2 Post–COVID-19 Interdisciplinary Clinical Care Network, Vancouver, British Columbia, Canada; 3 Centre for Clinical Epidemiology & Evaluation, Vancouver, British Columbia, Canada; 4 Department of Psychiatry, Massachusetts General Hospital, Boston, Massachusetts; 5 Department of Psychiatry, Harvard Medical School, Boston, Massachusetts

## Abstract

**Importance:**

Psychiatric symptoms are reportedly common among adults with post–COVID-19 condition (PCC). However, nationally representative data regarding symptom prevalence, treatment uptake, and barriers to care are needed to inform the development of care models.

**Objectives:**

To evaluate the prevalence of psychiatric symptoms in US adults with PCC compared with those without PCC and assess treatment uptake and cost-related barriers to treatment.

**Design, Setting, and Participants:**

Data from the 2022 National Health Interview Survey (NHIS), a nationally representative US cross-sectional survey, were analyzed between October 2023 and February 2024.

**Exposure:**

Current PCC, defined as new symptoms following SARS-CoV-2 infection lasting more than 3 months and ongoing at the time of interview.

**Main Outcomes and Measures:**

Depression symptoms were evaluated by the Patient Health Questionnaire-8 and anxiety symptoms were assessed using the General Anxiety Disorder-7 instrument. Participants were classified as having received treatment if they received mental health counseling or therapy or medications for mental health. Sleep difficulties, cognitive difficulties, disabling fatigue, and cost-related barriers were assessed from additional NHIS questions.

**Results:**

Of the 25 122 participants representing approximately 231 million US adults (median [IQR] age, 46 [32-61] years; 49.8% male and 50.2% female participants), a weighted prevalence (wPr) of 3.4% (95% CI, 3.1%-3.6%) had current PCC. Compared with other US adults, participants with current PCC were more likely to have depression symptoms (wPr, 16.8% vs 7.1%; adjusted odds ratio [AOR], 1.96; 95% CI, 1.51-2.55), anxiety symptoms (wPr, 16.7% vs 6.3%; AOR, 2.21; 95% CI, 1.53-3.19), sleep difficulties (wPr, 41.5% vs 22.7%; AOR 1.95; 95% CI, 1.65-2.29), cognitive difficulties (wPr, 35.0% vs 19.5%; AOR, 2.04; 95% CI, 1.66-2.50), and disabling fatigue (wPr, 4.0% vs 1.6%; AOR, 1.85; 95% CI, 1.20-2.86). Among participants who had depression or anxiety symptoms, those with PCC had a similar likelihood of not having received treatment (wPr, 28.2% vs 34.9%; AOR, 1.02; 95% CI, 0.66-1.57). However, participants with current PCC were more likely to report a cost-related barrier to accessing mental health counseling or therapy (wPr, 37.2% vs 23.3%; AOR, 2.05; 95% CI, 1.40-2.98).

**Conclusions and Relevance:**

The findings of this study suggest that people with PCC have a higher prevalence of psychiatric symptoms than other adults but are more likely to experience cost-related barriers to accessing therapy. Care pathways for PCC should consider prioritizing mental health screening and affordable treatment.

## Key Points

**Question:**

What is the prevalence of psychiatric symptoms in US adults with post–COVID-19 condition (PCC) compared with those without PCC?

**Findings:**

In this nationally representative cross-sectional study of 25 122 participants, those experiencing PCC were approximately twice as likely to report depression and anxiety symptoms than other US adults. Among individuals with these symptoms, adults with PCC were just as likely to have received mental health treatment but more likely to report cost-related barriers to accessing therapy.

**Meaning:**

The results of this study suggest that psychiatric symptoms are relatively common in people with PCC and that more affordable care is needed for this population.

## Teaser

This cross-sectional study examines the prevalence of psychiatric illness in individuals with post–COVID-19 condition, their receipt of treatment, and the cost-related barriers they encounter.

## Introduction

Recent estimates indicate that more than 15 million US adults have experienced post–COVID-19 condition (PCC).^1,2^ Also known as long COVID, PCC is currently defined by the World Health Organization as persistent symptoms occurring at least 3 months following a SARS-CoV-2 infection.^3^ People with PCC can experience long-term impairment in their quality of life and occupational functioning.^4,5,6,7,8^ Among the hundreds of symptoms described by people with PCC, psychiatric symptoms are prominent.^9^ Psychiatric symptoms frequently reported by these individuals have included depression, anxiety, sleep difficulties, fatigue, cognitive difficulties, and posttraumatic stress.^10,11,12^

Although it is widely recognized that these psychiatric symptoms are common in PCC, establishing reliable estimates of their prevalence has been difficult. Numerous studies have reported high rates of patient-reported psychiatric symptoms in PCC.^5,9,13,14,15,16,17,18^ However, as highlighted in a recent report by the US Substance Abuse and Mental Health Services Administration and in several academic commentaries, much of this research is limited by small sample sizes, selection biases, inconsistent definitions of PCC, absence of SARS-CoV-2 uninfected controls, or the underrepresentation of disadvantaged groups.^19,20,21,22,23^ An incomplete understanding of the prevalence of psychiatric symptoms in PCC has hindered health systems from fully appreciating the resources required to support mental health in this population.

The mental health treatment uptake among people with PCC also warrants further investigation. Studies using health administrative databases have observed that individuals are more likely to be diagnosed with psychiatric illness and receive psychiatric treatment in the months following SARS-CoV-2 infection.^24,25,26,27^ However, these studies do not focus on people with PCC and do not capture the experiences of individuals who were symptomatic but did not seek care for their symptoms. The experiences of individuals who were unable to access care due to costs, stigma, or other reasons are important to consider when developing PCC-focused mental health supports. Adults with mental illness frequently experience barriers to care and may be underserved—a problem that was exacerbated during the COVID-19 pandemic.^28,29^

To develop accessible care pathways for people with PCC who have psychiatric symptoms, we require more inclusive estimates of the prevalence of these symptoms in this population and an understanding of whether symptomatic individuals have been able to access care. In this nationally representative cross-sectional study, we evaluated the prevalence of psychiatric symptoms, uptake of mental health treatment, and barriers to treatment in people with PCC.

## Methods

### Study Design

We analyzed data from the National Health Interview Survey (NHIS), a nationwide cross-sectional survey of the noninstitutionalized US population conducted annually by the National Center for Health Statistics (NCHS). The NHIS is conducted 12 months of the year and applies geographically clustered sampling techniques to achieve nationally representative samples.^30^ Consenting participants are interviewed in their homes or by telephone by trained field representatives. If a participant is physically or cognitively disabled, a knowledgeable proxy can answer questions on their behalf. We used data from the 2022 iteration of the NHIS, which was the first year that PCC questions were included.^2,30^ The 2022 survey also contained some questions related to depression, anxiety, sleep, and fatigue, which are not included annually. The response rate for the 2022 survey was 47.7%. Data were analyzed between October 2023 and February 2024. The University of British Columbia Research Ethics Board deemed this study exempt from review because it involved publicly available data. We followed the Strengthening the Reporting of Observational Studies in Epidemiology (STROBE) reporting guideline.

### Definition of Exposure

The primary exposure was the presence of symptomatic PCC at the time of the interview (ie, current PCC). Using the approach established by the NCHS,^2^ respondents were classified as having current PCC if they reported a history of SARS-CoV-2 infection (a prior positive test or if informed by a health professional), had new symptoms following infection that lasted greater than 3 months, and had symptoms at the time of the interview (eMethods in Supplement 1). The control group included all other US adults.

### Definitions of Outcomes

#### Psychiatric Symptoms

Depression and anxiety were our primary symptoms of interest. Our prespecified outcome measure for depression symptoms was the presence of at least moderate depression indicated by a score of 10 or higher on the 8-item Patient Health Questionnaire (PHQ-8).^31^ The PHQ-8 is scored from 0 to 24 and asks participants about depressive disorder symptoms experienced in the prior 2 weeks. A score of 10 or higher on the PHQ-8 suggests clinically significant depression and was associated with impairment in multiple health-related quality-of-life domains in a nationwide study. The PHQ-8 is identical to the PHQ-9 but excludes the item that asks about thoughts of self-harm. The PHQ-8 and PHQ-9 have been shown to have comparable sensitivity and specificity for identifying clinical depression.^32,33^ Our prespecified outcome measure for anxiety symptoms was the presence of at least moderate anxiety indicated by a score of 10 or higher on the 7-item General Anxiety Disorder (GAD-7) scale.^34^ The GAD-7 is scored from 0 to 21 and asks participants about anxiety disorder symptoms experienced in the prior 2 weeks. A score of 10 or higher on the GAD-7 suggests the presence of generalized anxiety disorder and is associated with increased disability days and reduced health-related quality of life.

We examined the presence of sleep difficulties, cognitive difficulties, and disabling fatigue as secondary outcomes. Participants were classified as having sleep difficulties if they reported having difficulty falling asleep or staying asleep most days or every day in the past 30 days.^35,36^ The NHIS contains a question from the Washington Group (WG) that asks participants if they have difficulty remembering or concentrating; we classified participants as having cognitive difficulties if they responded that they had at least some difficulty.^37,38^ This question was shown to have a sensitivity of 83% for identifying at least mild cognitive impairment in a population-based sample.^39^ Fatigue was assessed using 3 WG questions that asked about the frequency, duration, and severity of tiredness. We used syntax recommended by the WG to assign an indicator score from 1 to 4 based on the combination of responses.^38^ Consistent with prior work, participants were classified as having disabling fatigue if their indicator score was 4.^40,41^

#### Uptake of Mental Health Treatment

The uptake of any mental health treatment was a primary outcome. Use of medication and receipt of mental health counseling or therapy were analyzed separately as secondary outcomes. Following previously described methods,^42,43^ we classified study participants as having received psychiatric treatment if they reported prescription medication use or receipt of mental health counseling or therapy in the preceding 12 months. Prescription medication use was determined based on an affirmative response to at least 1 of 3 questions. The first 2 of the questions asked about whether the participant was taking a medication for depression and anxiety. A third question asked whether they took medication in the last 12 months for other emotions. All participants were asked if they had received counseling or therapy from a mental health professional in the preceding 12 months. A mental health professional was specified to the participant as a psychiatrist, psychologist, psychiatric nurse, or clinical social worker.

#### Cost-Related Barriers

We evaluated the presence of a cost-related barrier to mental health counseling or therapy as a primary outcome. The NHIS participants were asked if, in the preceding 12 months, they delayed getting counseling or therapy from a mental health professional because of the cost and if there was any time when they needed counseling or therapy from a mental health professional but did not obtain it because of the cost. We classified participants as having a cost-related barrier if they responded affirmatively to either question.

### Statistical Analysis

We prespecified covariates for our statistical models based on the clinical expertise of our team and literature review.^2,15,26,44,45,46,47,48,49,50,51,52,53,54,55,56^ These included NHIS-related variables and potential sociodemographic and clinical confounders, including interview month, US region, sex, age, urban-rural classification,^57^ race and ethnicity, educational level, marital/partner status, employment status, income relative to federal poverty line, current smoking status, health insurance, functional disability, number of medical comorbidities, and number of COVID-19 vaccinations (covariate definitions outlined in the eMethods in Supplement 1). Race and ethnicity were included in the analysis because the prevalence of PCC and access to mental health care may differ across different racial groups.^58,59^ The NHIS participants were asked whether they had been previously diagnosed with depression or anxiety by a clinician but not when they were diagnosed. As we were unable to determine whether the diagnosis referred to current symptoms or prior episodes, we did not adjust for this factor.

We assessed the association between current PCC and each outcome by conducting bivariate logistic regression analyses followed by multivariable logistic regression that included the prespecified covariates. Among the subgroup of participants classified as experiencing symptoms of at least moderate depression or anxiety, we then evaluated the proportion of individuals with current PCC who had mental health treatment uptake and the proportion with cost-related barriers.

Analyses were conducted in SAS Studio (SAS Institute Inc). We applied NHIS survey weights using the *proc survey* functions in SAS to generate prevalence and regression estimates representative of the US population with corresponding CIs accounting for the complex sampling design. Statistical significance was 2-sided, and the significance threshold was set at *P* < .01 based on Bonferroni correction for 4 primary outcomes. Missing income data were imputed by the NCHS.^30^ For respondents who had only 1 item missing for PHQ-8 or GAD-7, the NCHS imputes the mean value of the completed items such that a total score can still be calculated.^30^ We otherwise conducted complete case analysis. Records with incomplete or missing data for the exposure, covariates, or primary outcomes were excluded.

We conducted 6 sets of sensitivity analyses. First, we assessed the robustness of the results by repeating analyses with only a 5% random sample of the much larger control group. Second, we evaluated different severity thresholds for depression (PHQ-8 ≥ 5; PHQ-8 ≥ 15) and anxiety (GAD-7 ≥ 5; GAD-7 ≥ 15). Third, we used the WG depression and anxiety measures (eMethods in Supplement 1). These measures do not specify a time period for symptoms and instead inquire about frequency from daily to a few times a year. Fourth, we conducted analyses comparing outcomes in participants with PCC with other COVID-19 survivors vs all other US adults. This explored whether findings were specific to PCC rather than a consequence of COVID-19. Fifth, we broadened the subgroup analyses to include all participants who reported symptoms of depression or anxiety at least a few times a year. This permitted inclusion of respondents who may have been symptomatic previously but not within the past 2 weeks. Sixth, we restricted analysis of treatment uptake to participants who were currently taking medications for anxiety or depression. This excluded individuals who may have been treated prior to their recent symptoms but were no longer receiving treatment.

## Results

### Characteristics of Study Population

There were 25 122 participants included in the analyses representing 231 190 640 (95% CI, 223 886 954-238 494 326) US adults. The median age was 46 (IQR, 32-61) years; there were 49.8% male and 50.2% female participants. A weighted prevalence (wPr) of 39.9% (95% CI, 39.1%-40.6%) had a history of COVID-19, and of participants who experienced symptomatic acute COVID-19, 19.3% (95% CI, 18.3%-20.4%) developed PCC (Figure; eFigure in Supplement 1). Of the total included participants, an estimated 3.4% (95% CI, 3.1%-3.6%) or 7 792 381 (95% CI, 7 136 381-8 447 830) were classified as having current PCC. Compared with other US adults, participants with current PCC were more likely to be female, non-Hispanic White, and have multiple comorbidities but less likely to have been vaccinated (Table 1).

**Figure.**
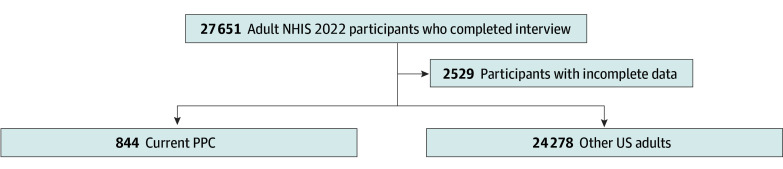
Participant Flow Diagram Data were considered incomplete if there were missing values for any of the outcome measures or covariates used in the multivariable models. The weighted percentages for each group were calculated by using the weighted population value from the prior level on the flow diagram as the denominator: weighted percentages in participants with incomplete data, 9.5 (95% CI, 9.0-9.9); complete data, 90.5 (95% CI, 90.1-91.0); current post–COVID-19 condition (PCC), 3.4 (95% CI, 3.1-3.6); and other US adults, 96.6 (95% CI, 96.4-96.9). The eMethods provides further details regarding variable definitions and the eFigure provides a more detailed flow diagram in Supplement 1. NHIS indicates National Health Interview Survey.

**Table 1.** Characteristics of US Adults With and Without Current PCC^a^

Characteristic	Current PCC^b^		Other US adults^c^	
	Unweighted, No.	Weighted % (95% CI)	Unweighted, No.	Weighted % (95% CI)
Total (row %)	844	3.4 (3.1-3.6)	24 278	96.6 (96.4-96.9)
Interview month (2022)				
January-March	182	21.7 (18.3-25.0)	5923	24.9 (24.1-25.6)
April-June	197	25.4 (21.9-29.0)	6057	25.0 (24.3-25.7)
July-September	235	26.8 (23.2-30.4)	6269	25.0 (24.4-25.7)
October-December	230	26.1 (22.8-29.4)	6029	25.1 (24.4-25.9)
Sex				
Female	570	66.2 (62.5-70.0)	13 000	50.4 (49.6-51.1)
Male	274	33.8 (33.0-37.5)	11 278	49.6 (48.9-50.4)
Age, median (IQR), y^d^		46 (35-58)		46 (31-62)
Age category, y				
18-34	150	22.7 (19.1-26.3)	5070	29.0 (28.2-29.9)
35-49	262	34.6 (30.8-38.5)	5343	24.0 (23.4-24.6)
50-64	239	27.7 (24.2-31.2)	6047	24.6 (24.0-25.2)
≥65	193	15.0 (12.6-17.4)	7818	22.4 (21.7-23.0)
US region				
Northeast	114	14.9 (11.7-18.1)	3973	17.3 (16.3-18.4)
Midwest	208	21.4 (18.4-24.5)	5336	20.9 (19.8-22.0)
South	323	38.7 (34.6-42.8)	8913	38.0 (36.4-39.6)
West	199	24.9 (21.1-28.8)	6056	23.7 (22.2-25.2)
Urban-rural classification^e^				
Large central metropolitan	182	24.0 (20.2-27.8)	7264	30.8 (28.6-33.0)
Large fringe metropolitan	185	23.9 (20.1-27.6)	5669	25.2 (22.9-27.5)
Medium and small metropolitan	299	34.4 (29.8-39.0)	7541	30.0 (27.2-32.8)
Nonmetropolitan	178	17.7 (14.6-20.9)	3804	13.9 (12.9-15.0)
Race and ethnicity				
Asian and other^f^	44	5.5 (3.6-7.4)	2096	9.0 (8.2-9.8)
Hispanic	123	17.5 (13.9-21.0)	3402	16.9 (15.6-18.3)
Non-Hispanic Black	62	7.3 (5.3-9.3)	2642	11.4 (10.6-12.3)
Non-Hispanic White	615	69.8 (65.7-73.9)	16 138	62.6 (61.1-64.2)
More than high school or GED education	576	66.4 (62.4-70.4)	16 167	62.6 (61.6-63.6)
Married or with partner	479	66.4 (63.0-69.7)	12 926	60.6 (59.8-61.4)
Employed	543	67.9 (64.4-71.5)	14 126	63.8 (63.0-64.5)
Income ≥200% FPL^g^	596	72.3 (68.6-76.0)	17 743	73.1 (72.1-74.1)
Health insurance	794	92.8 (90.7-95.0)	22 494	90.5 (89.8-91.1)
Current smoker	99	11.4 (9.0-13.8)	2822	11.5 (11.0-12.0)
Functional disability^h^	145	14.8 (12.1-17.4)	2368	8.7 (8.2-9.1)
No. of medical comorbidities^i^				
0	159	20.4 (17.0-23.7)	7105	33.8 (32.9-34.6)
1-2	375	45.7 (41.9-49.6)	10 362	42.9 (42.1-43.6)
≥3	310	33.9 (30.0-37.8)	6811	23.4 (22.7-24.1)
COVID-19 vaccination				
0	228	25.8 (22.4-29.3)	4651	20.3 (19.4-21.1)
1	62	7.4 (5.3-9.4)	1037	4.4 (4.1-4.7)
≥2	616	66.8 (63.2-70.4)	20 447	75.4 (74.5-76.3)
Acute COVID-19 severity^j^				
Never had COVID-19	NA	NA	15 814	62.2 (61.4-63.0)
Asymptomatic	NA	NA	1017	4.2 (3.8-4.6)
Mild symptoms	162	18.3 (15.2-21.4)	3404	15.3 (14.7-15.9)
Moderate symptoms	346	43.2 (39.0-47.4)	3016	13.8 (13.2-14.3)
Severe symptoms	336	38.5 (34.4-42.6)	1027	4.5 (4.2-4.8)

Abbreviations: FPL, federal poverty line; GED, General Educational Development; NA, not applicable; PCC, post–COVID-19 condition.

^a^ Sample population is the crude number of participants; weighted population is the corresponding US adult population after applying National Health Interview Survey (NHIS) survey weights. The weighted percentages for each group were calculated by using the weighted population value.

^b^ Weighted No., 7 792 106 (95% CI, 7 136 381-8 447 830).

^c^ Weighted No., 223 398 534 (95% CI, 216 289 409-230 507 659).

^d^ Excludes participants aged 85 years or older due to top coding; the median was derived from the sample for current PCC (n = 830) and other US adults (n = 23 415).

^e^ Based on National Center for Health Statistics (NCHS) rural-urban classification scheme for counties.^57^

^f^ Other race includes Native American and Alaska Native, multiracial, and other groups.

^g^ For respondents who do not report income, this is estimated in the NHIS using imputation; income data are then used to determine ratio above the FPL.

^h^ Respondents were classified as having functional disability if they responded a lot of difficulty or cannot do at all to at least 1 of the questions on the Washington Group Set Disability Indicator.

^i^ Self-reported medical comorbidities included hypertension ever, high cholesterol ever, coronary artery disease ever, asthma currently, diabetes ever, chronic obstruct pulmonary disease ever, arthritis ever, cancer ever, and obesity currently.

^j^ Respondents were classified as having a history of COVID-19 if they reported a prior positive test or diagnosis by a health professional.

### Psychiatric Symptoms

After adjustment for covariates, participants with current PCC were more likely to report at least moderate symptoms of depression (wPr, 16.8% vs 7.1%; adjusted odds ratio [AOR], 1.96; 95% CI, 1.51-2.55) and anxiety (wPr, 16.7% vs 6.3%; AOR, 2.21; 95% CI, 1.53-3.19) than other US adults (Table 2; eTable 1 in Supplement 1). Participants with current PCC were more likely than other US adults to report sleep difficulties (wPr, 41.5% vs 22.7%; AOR, 1.95; 95% CI, 1.65-2.29), cognitive difficulties (wPr, 35.0% vs 19.5%; AOR, 2.04; 95% CI, 1.66-2.50), and disabling fatigue (wPr, 4.0% vs 1.6%; AOR, 1.85; 95% CI, 1.20-2.86). In sensitivity analyses, the findings were similar when using a 5% random sample of the control group (eTable 2 in Supplement 1), across different severity thresholds for depression and anxiety symptoms (eTable 3 in Supplement 1), when using the WG depression and WG anxiety measures (eTable 4 in Supplement 1), and when comparing symptoms of participants with current PCC with those of other COVID-19 survivors (eTable 5 in Supplement 1).

**Table 2.** Psychiatric Symptoms in Participants With Current PCC Compared With Other US Adults^a^

Outcome	Weighted % (95% CI)		Bivariate, OR (95% CI)	Multivariable^b^
	Current PCC	Other US adults		AOR (95% CI)
At least moderate depression symptoms (PHQ-8 ≥ 10)	16.8 (13.8-19.7)	7.1 (6.6-7.5)	2.64 (2.11-3.30)	1.96 (1.51-2.55)
At least moderate anxiety symptoms (GAD-7 ≥ 10)	16.7 (13.5-19.8)	6.3 (5.9-6.8)	3.17 (2.30-4.37)	2.21 (1.53-3.19)
Sleep difficulties	41.5 (37.8-45.2)	22.7 (22.0-23.4)	2.42 (2.06-2.83)	1.95 (1.65-2.29)
Cognitive difficulties	35.0 (31.0-39.0)	19.5 (18.8-20.2)	2.22 (1.86-2.66)	2.04 (1.66-2.50)
Disabling fatigue	4.0 (2.5-5.4)	1.6 (1.4-1.8)	2.59 (1.72-3.89)	1.85 (1.20-2.86)

Abbreviations: AOR, adjusted odds ratio; GAD-7, 7-item General Anxiety Disorder; OR, odds ratio; PCC, post–COVID-19 condition; PHQ-8, 8-item Patient Health Questionnaire.

^a^ All multivariable models are adjusted for interview month, US region, sex, age, urban-rural classification, race and ethnicity, educational level, marital/partner status, employment status, income relative to federal poverty line, current smoking status, health insurance, functional disability, number of medical comorbidities, and number of COVID-19 vaccinations. Full results of the bivariate and multivariable models for the primary outcomes are reported in eTable 1 in Supplement 1.

^b^ All *P* values were <.001.

### Mental Health Treatment Uptake and Cost-Related Barriers

Of the participants with current PCC and symptoms of depression or anxiety, 28.2% vs 34.9% (1.02; 95% CI, 0.66-1.57) had not received treatment in the prior 12 months, and this was not significantly different than the proportion of other US adults who had not received treatment (wPr, 37.2% vs 23.3%; AOR, 2.05; 95% CI, 1.40-2.98) (Table 3; eTable 6 in Supplement 1). Of the participants with symptoms of depression or anxiety, those with current PCC were more likely to report a cost-related barrier to mental health counseling or therapy (AOR, 2.12; 95% CI, 1.65-2.73). In sensitivity analyses, including all participants with depression or anxiety symptoms at least a few times a year (eTable 7 in Supplement 1) and when analysis of treatment uptake was restricted to current medication use (eTable 8 in Supplement 1) resulted in similar findings.

**Table 3.** Associations Between Current PCC, Mental Health Treatment Uptake, and Cost-Related Barriers Among Participants With Depression or Anxiety Symptoms^a^

Outcome	Weighted % (95% CI)		Bivariate, OR (95% CI)	Multivariable	
	Current PCC	Other US adults		AOR (95% CI)	*P* value
No uptake of mental health treatment in past 12 mo	28.2 (20.2-36.3)	34.9 (32.5-37.3)	0.80 (0.54-1.18)	1.02 (0.66-1.57)	.94
Not treated with medication	35.4 (27.0-43.9)	44.0 (41.6-46.4)	0.80 (0.55-1.16)	0.94 (0.61-1.43)	.76
Not treated with counseling or therapy	49.6 (40.8-58.4)	59.3 (56.7-61.8)	0.68 (0.48-0.96)	0.70 (0.47-1.03)	.07
Cost-related barrier to mental health counseling or therapy in past 12 mo	37.2 (28.8-45.6)	23.3 (21.1-25.5)	1.96 (1.37-2.79)	2.05 (1.40-2.98)	<.001
Delayed treatment	31.8 (24.0-39.7)	20.8 (18.7-22.9)	1.48 (1.04-2.10)	1.93 (1.31-2.85)	<.001
Needed but did not receive treatment	33.6 (25.4-41.7)	20.7 (18.6-22.8)	1.89 (1.31-2.74)	1.96 (1.32-2.91)	<.001

Abbreviations: AOR, adjusted odds ratio; OR, odds ratio; PCC, post–COVID-19 condition.

^a^ The analyses include all participants who experienced at least moderate depression (8-item Patient Health Questionnaire score ≥10) or at least moderate anxiety (Generalized Anxiety Disorder-7 score ≥10) (n = 2328). All multivariable models are adjusted for interview month, US region, sex, age, urban-rural classification, race and ethnicity, educational level, marital/partner status, employment status, income relative to federal poverty line, current smoking status, health insurance, functional disability, number of medical comorbidities, and number of COVID-19 vaccinations. Full results of multivariable models for uptake of mental health treatment and cost-related barriers to counseling or therapy are reported in eTable 5 in Supplement 1.

## Discussion

In this nationally representative cross-sectional study, we found that depression and anxiety symptoms, sleep difficulties, cognitive difficulties, and disabling fatigue were all more prevalent in participants with current PCC than other US adults. Participants with current PCC experiencing depression or anxiety symptoms had a similar rate of treatment uptake as other US adults but were more likely to report cost-related barriers to mental health counseling or therapy.

This study builds on previous research that showed that patient-reported symptoms of depression and anxiety are relatively common in PCC.^16,17,18,44,49,60,61,62^ However, our prevalence estimates for depression symptoms (16.8%) and anxiety symptoms (16.7%) are lower than those in most other large studies.^16,17,18,44,61^ As these studies did not apply a complex survey method and were promoted as being focused on COVID-19, sampling may not have been fully representative and selection biases may have resulted in overestimates. It is also possible that participants in our study were surveyed later relative to their COVID-19 diagnoses and were thus less likely to be symptomatic.

More than a quarter of participants with current PCC who were experiencing psychiatric symptoms had not received treatment, and this represents an opportunity to improve access to mental health care in this population. With support from the US Department of Health and Human Services, health care systems are working to develop care pathways specific to PCC.^63^ These pathways can integrate mental health services by, for example, incorporating routine mental health screening in follow-up for individuals recovering from COVID-19 and including mental health professionals in multidisciplinary PCC clinics. In contexts in which mental health services are sparse, telehealth and group-based programs could be leveraged.^64^

Participants with PCC were more likely to report cost-related barriers to accessing counseling or therapy than other US adults. People with PCC may have more difficulty paying for counseling or therapy due to lost employment wages and greater costs of managing complications from COVID-19, or they may experience challenges obtaining health plan authorization for these supports.^4,50,65,66^ This finding may also be an indication that people with PCC are seeking counseling or therapy more frequently because they find it beneficial. In a recent randomized clinical trial, cognitive behavioral therapy was more effective than usual care for treating fatigue in individuals recovering from COVID-19.^67^ Further research should investigate counseling or therapy as a treatment strategy in PCC and determine how it can be delivered affordably.

However, when developing and delivering treatment programs, clinicians should be cognizant that people with PCC with or without psychiatric symptoms may be apprehensive about accessing care. These individuals have described experiencing stigma and medical gaslighting from clinicians, sometimes being told that their physical symptoms are psychosomatic.^53,65,66,68,69,70^ Clinicians must recognize that, although depression and anxiety are more common in PCC, these symptoms are only present in a minority of individuals (<25% in our study). Most individuals with PCC do not have substantial psychiatric symptoms, and psychiatric and physical symptoms must be evaluated without any preconceived notions or assumptions. Standardized screening strategies for psychiatric symptoms in PCC clinics may help normalize mental health assessments for this population.^64^

### Strengths and Limitations

This study has several strengths. The interview-based, nationwide, complex sampling design increases confidence that the reported prevalence estimates are reliable and representative. Due to its robust methods, NHIS data have been used for decades to monitor symptom and disease prevalence, behaviors, and health care access in the US population.^30^ We used the PHQ-8 and GAD-7 to measure depression and anxiety symptoms; these instruments are well validated, widely used, and correspond well with clinically important outcomes.^31,32,33,34^ Our sensitivity analyses including different symptom severity thresholds, measurement instruments, symptom timelines, and comparison groups all further support the validity of our findings.

Our study was also able to investigate mental health treatment uptake and cost-related barriers in the PCC population. Because NHIS sampling is population based and not limited to a particular health system, we expect that the study was more inclusive of individuals with economic disadvantages and those who had not sought care. These groups of individuals may not have been captured in studies involving health administrative data and convenience sampling from online platforms or PCC clinics.

This study has limitations. First, as a cross-sectional study, we were unable to make any causal inferences regarding psychiatric symptoms and PCC. It has been challenging for researchers to fully understand whether psychological distress is a risk factor for PCC, a consequence of, or both^15,71^; our study did not attempt to address this issue. Second, it was not possible for us to determine the timing or duration of participants’ psychiatric symptoms relative to COVID-19 diagnosis because the dates of infection and symptom onset are not captured by the NHIS. Similarly, we were unable to appreciate when participants may have received mental health treatment and whether some had received treatment and subsequently recovered. Third, our assessment of treatment uptake was limited to prescription medication and counseling or therapy by a mental health professional. Other treatments, such as self-directed cognitive behavioral therapy, were not captured. Fourth, there were likely unmeasured confounders that contributed to the difference between groups. For example, psychiatric history and the total number of SARS-CoV-2 infections were not accounted for in the study. Fifth, all data were self-reported. Therefore, COVID-19 status could not be confirmed, we were unable to adjust for detailed comorbidity information, and responses were subject to recall bias.

## Conclusions

In summary, this nationally representative cross-sectional study observed that psychiatric symptoms are more common in people with PCC than in other US adults, but many individuals with PCC do not receive mental health treatment and are more likely to experience cost-related barriers. At the individual level, clinicians should be aware that people with PCC are about twice as likely to report psychiatric symptoms and consider routine screening for these symptoms. At the health system level, it may be useful for health care leaders to prioritize the inclusion of affordable mental health supports when designing care pathways for PCC.

## Notes

Supplement 1. **eMethods.** Detailed Methods **eFigure.** Detailed Participant Flow Diagram **eTable 1.** Bivariate and Full Multivariable Models for Psychiatric Symptoms **eTable 2.** Associations Between Current PCC and Psychiatric Symptoms Using a 5% Sample of the Control Group (Sensitivity Analyses) **eTable 3.** Associations Between Current PCC and Symptoms of Depression and Anxiety Using Different Severity Thresholds (Sensitivity Analyses) **eTable 4.** Association Between Current PCC and Psychiatric Symptoms Measured Using WG-DEP and WG-ANX (Sensitivity Analyses) **eTable 5.** Psychiatric Symptoms in Participants With Current PCC Compared to Other COVID-19 Survivors (Sensitivity Analyses) **eTable 6.** Bivariate and Full Multivariable Models for Mental Health Treatment Uptake and Cost-Related Barriers Among Participants With Depression or Anxiety Symptoms **eTable 7.** Associations Between Current PCC, Mental Health Treatment Uptake and Cost-Related Barriers Among Participants With Depression or Anxiety Symptoms at Least “a Few Times a Year” (Sensitivity Analyses) **eTable 8.** Association Between Current PCC and Mental Health Treatment Uptake (Restricted to Current Medications Only) Among Participants With Depression or Anxiety Symptoms (Sensitivity Analyses)
eReferences

Supplement 2.
Data Sharing Statement
