# Supplementary material for: Psychiatric Symptoms, Treatment Uptake, and Barriers to Mental Health Care Among US Adults With Post–COVID-19 Condition
Source: JAMA Netw Open. 2024 Apr 25;7(4):e248481. doi: 10.1001/jamanetworkopen.2024.8481 (PMC11046346; doi:10.1001/jamanetworkopen.2024.8481)
Supplement: Supplement 1. — eMethods. Detailed Methods eFigure. Detailed Participant Flow Diagram eTable 1. Bivariate and Full Multivariable Models for Psychiatric Symptoms eTable 2. Associations Between Current PCC and Psychiatric Symptoms Using a 5% Sample of the Control Group (Sensitivity Analyses) eTable 3. Associations Between Current PCC and Symptoms of Depression and Anxiety Using Different Severity Thresholds (Sensitivity Analyses) eTable 4. Association Between Current PCC and Psychiatric Symptoms Measured Using WG-DEP and WG-ANX (Sensitivity Analyses) eTable 5. Psychiatric Symptoms in Participants With Current PCC Compared to Other COVID-19 Survivors (Sensitivity Analyses) eTable 6. Bivariate and Full Multivariable Models for Mental Health Treatment Uptake and Cost-Related Barriers Among Participants With Depression or Anxiety Symptoms eTable 7. Associations Between Current PCC, Mental Health Treatment Uptake and Cost-Related Barriers Among Participants With Depression or Anxiety Symptoms at Least “a Few Times a Year” (Sensitivity Analyses) eTable 8. Association Between Current PCC and Mental Health Treatment Uptake (Restricted to Current Medications Only) Among Participants With Depression or Anxiety Symptoms (Sensitivity Analyses) eReferences [file jamanetwopen-e248481-s001.pdf]

Supplementary Online Content

Naik H, Tran KC, Staples JA, Perlis RH, Levin A. Psychiatric symptoms, treatment uptake, and barriers to mental health care among US adults with post–COVID-19 condition. *JAMA Netw Open*. 2024;7(4):e248481. doi:10.1001/jamanetworkopen.2024.8481

eMethods. Detailed Methods

eFigure. Detailed Participant Flow Diagram

eTable 1. Bivariate and Full Multivariable Models for Psychiatric Symptoms

eTable 2. Associations Between Current PCC and Psychiatric Symptoms Using a 5% Sample of the Control Group (Sensitivity Analyses)

eTable 3. Associations Between Current PCC and Symptoms of Depression and Anxiety Using Different Severity Thresholds (Sensitivity Analyses)

eTable 4. Association Between Current PCC and Psychiatric Symptoms Measured Using WG-DEP and WG-ANX (Sensitivity Analyses)

eTable 5. Psychiatric Symptoms in Participants With Current PCC Compared to Other COVID-19 Survivors (Sensitivity Analyses)

eTable 6. Bivariate and Full Multivariable Models for Mental Health Treatment Uptake and Cost-Related Barriers Among Participants With Depression or Anxiety Symptoms

eTable 7. Associations Between Current PCC, Mental Health Treatment Uptake and Cost-Related Barriers Among Participants With Depression or Anxiety Symptoms at Least “a Few Times a Year” (Sensitivity Analyses)

eTable 8. Association Between Current PCC and Mental Health Treatment Uptake (Restricted to Current Medications Only) Among Participants With Depression or Anxiety Symptoms (Sensitivity Analyses)

eReferences

This supplementary material has been provided by the authors to give readers additional information about their work.

© 2024 Naik H et al. *JAMA Network Open.*

eMethods. Detailed Methods

1. Classification of Post-COVID-19 Condition

Participants were classified as having had COVID-19 in the past if they answered affirmatively to at least one of the following questions:

> *Has a doctor or other health professional ever told you that you had or likely had coronavirus or COVID-19?*
>
> *Did you ever take a test that showed you had coronavirus or COVID-19?*

Respondents classified as having COVID-19 were subsequently asked regarding the severity of their symptoms:

> *How would you describe your coronavirus symptoms when they were at their worst? Would you say no symptoms, mild symptoms, moderate symptoms, or severe symptoms?*

Participants who responded indicating that they had mild, moderate or severe symptoms (i.e. were not asymptomatic), were subsequently asked the following questions:

> *Did you have any symptoms lasting 3 months or longer that you did not have prior to having coronavirus or COVID-19?*
>
> *Do you have symptoms NOW?*

We classified respondents who answered affirmatively as having had symptoms lasting 3 months as having a history of post-COVID-19 condition (PCC). Participants who also indicated that they currently had symptoms were classified as having current PCC.[1] This is consistent with the current World Health Organization definition of post-COVID-19 condition.[2]

2. Covariate Definitions

Covariate	Question(s) in NHIS Survey	Classification
**Interview Month (2022)**	N/A	1. January, February, March<br>2. April, May, June<br>3. July, August, September<br>4. October, November, December
**Sex**	*Are you male or female?*	1. Male<br>2. Female
**Age**	N/A	Continuous variable categorized as follows:<br>1. 18-34<br>2. 35-49<br>3. 50-64<br>4. ≥65
**US Region**	N/A	Geographic region of respondent's home:<br>1. Northeast<br>2. Midwest<br>3. South<br>4. West

© 2024 Naik H et al. *JAMA Network Open.*

**Urban-rural classification**	N/A	Urbanicity of participant's county (or county-equivalent) provided directly by NCHS and categorized based on 2013 NCHS Urban-Rural classification scheme as follows:<br>1. Large central metro: in metropolitan statistical areas (MSA) of 1 million or more residents that 1) contain the entire population of the largest principal city of the MSA, or 2) are completely contained in the largest principal city of the MSA, or 3) contain at least 250,000 residents of any principal city of the MSA.<br>2. Large fringe metro: in MSAs of 1 million or more population that do not qualify as large central.<br>3. Medium and small metro: in MSAs of less than 1 million population.<br>4. Nonmetropolitan: in micropolitan statistical areas and non-core counties.
**Race and ethnicity**	Based on responses to two questions:<br>*1. Do you consider yourself to be Hispanic or Latino?*<br>2. *What race do you consider yourself to be?*	1. Non-Hispanic White<br>2. Non-Hispanic Black<br>3. Hispanic or Latino<br>4. Asian and other (includes 'Asian'; 'American Indian', 'Alaska Native'; 'Native Hawaiian'; 'Other Pacific Islander'; 'some other race')
**Education level**	*What is the HIGHEST level of school you completed or the highest degree you have received?*	1. HS, GED or less (includes 'Never attended/kindergarten only'; 'Grade 1-11'; '12th grade'; 'no diploma'; 'GED or equivalent'; 'High School Graduate')<br>2. More than HS or GED (includes 'some college, no degree'; 'associate degree: occupational, technical, or vocational program'; 'associate degree: academic program'; 'bachelor's degree'; 'master's degree'; 'professional school degree'; 'doctoral degree')
**Marital/ partner status**	*Are you now married, living with a partner together as an unmarried couple, or neither?*	1. Married or with partner (includes 'married'; 'living with a partner together as an unmarried couple')<br>2. Not married or with partner (if answer is 'Neither')
**Employment status**	Based on answers to the following questions:<br>*1. LAST WEEK, did you work for pay at a job or business?*<br>*2. Did you have a job or business LAST WEEK, but were temporarily absent due to illness, vacation, family or maternity leave, or some other reason?*	1. Employed (if worked in the last week, working in the last 12 months but did not work last week due to temporary absence, seasonal/contract work, or worked last week at family-owned job or business not for pay)<br>2. Not employed (if not satisfying criteria for employed)

© 2024 Naik H et al. *JAMA Network Open.*

	3. *What is the MAIN reason you were not working for pay at a job or business last week?*<br>4. *When was the last time you worked for pay at a job or business, even if only for a few days?*	
**Income**	*The next questions are about your total FAMILY income in LAST YEAR BEFORE TAXES. What is your best estimate of TOTAL INCOME from all sources, before taxes, in LAST YEAR?*<br><br>Participants who decline to respond are provided the opportunity to indicate their income within predetermined brackets and relative to the federal poverty line (FPL).	For participants with missing responses to family income, this is imputed by NCHS based on responses to the bracket responses and other survey responses. The estimated family income to FPL ratio is then calculated. FPL is estimated based on the forecasted federal poverty threshold for the participant's family size and age. In this study, family income was categorized as follows:<br>1. <200% FPL<br>2. ≥200% FPL
**Health insurance**	*What kinds of health insurance or health care coverage do you have? Is it...Private health insurance, Medicare, Medicare supplement, Medicaid, Children's Health Insurance Program or CHIP, military related health care including TRICARE, CHAMPUS, VA health care and CHAMP-VA, Indian Health Service, a state-sponsored health plan, or an other government program*?	Based on the combination of insurance plans indicated, NCHS classifies participants as "not covered" or "covered":<br>1. No health insurance (i.e. "not covered" if they did not have private health insurance, Medicare, Medicaid, CHIP, a state-sponsored health plan, other government plan or military health plan)<br>2. Health insurance (i.e. "covered")
**Smoking status**	*Do you NOW smoke cigarettes every day, some days or not at all?*	1. Current smoker (includes 'current every day smoker' or 'current some day smoker')<br>2. Not current smoker (includes 'former smoker' and 'never smoker')
**Functional disability**	Six questions from the Washington Group Short Set (WG-SS) Disability Indicator:<br>1. **Vision**: *Do you have difficulty seeing even if wearing glasses?*<br>2. **Communication**: *Using your usual language, do you have difficulty communicating, for example, understanding or being understood?*<br>3. **Hearing**: *Do you have difficulty hearing even if using a hearing aid?*<br>4. **Cognition**: *Do you have difficulty remembering or concentrating?*<br>5. **Self-care**: *Do you have difficulty with self-care, such as washing all over or dressing?*<br>6. **Mobility**: *Do you have difficulty walking or climbing stairs?*	1. Functional disability (if responds "a lot of difficulty" or "cannot do at all" to any of the six questions on WG-SS)<br>2. No functional disability
**No. of medical comorbidities**	The presence of particular comorbidities was ascertained from the following questions:	The total number of comorbidities was counted and categorized:<br>1. 0<br>2. 1-2

© 2024 Naik H et al. *JAMA Network Open.*

	1. **Hypertension**: *Have you EVER been told by a doctor or other health professional that you had hypertension, also called high blood pressure?*<br>2. **High cholesterol**: *Have you EVER been told by a doctor or other health professional that you had high cholesterol?*<br>3. **Coronary artery disease:** *Have you EVER been told by a doctor or other health professional that you had coronary heart disease?*<br>4. **Current asthma:** *Have you EVER been told by a doctor or other health professional that you had asthma? Do you still have asthma?*<br>5. **Diabetes**: *Has a doctor or other health professional EVER told you that you had diabetes?*<br>6. **COPD:** *Have you EVER been told by a doctor or other health professional that you had Chronic Obstructive Pulmonary Disease, C.O.P.D., emphysema, or chronic bronchitis?*<br>7. **Arthritis:** *Have you EVER been told by a doctor or other health professional that you had some form of arthritis, rheumatoid arthritis, gout, lupus, or fibromyalgia (fy-bro-my-AL-jeeuh)?*<br>8. **Obesity:** *How tall are you without shoes? How much did you weigh before your pregnancy (if pregnant)?/How much do you weigh? Classified as obese if body mass index ≥30 kg/m²*.	3. 3 or more
**COVID-19 vaccinations**	*How many COVID-19 vaccinations have you received?*	1. 0<br>2. 1<br>3. ≥2

3. Measures of Depression and Anxiety Symptoms for Sensitivity Analyses

In sensitivity analyses, we used the 2-item depression question set developed by the Washington Group on Disability Statistics (WG-DEP) to assess depression, and the analogous 2-item anxiety question set (WG-ANX) to measure anxiety. Using these measures, a respondent can be classified as having at least medium levels of depression or anxiety if they report symptoms occurring at least weekly, and the intensity of the symptoms being "between a little and a lot" or "a lot".[3,4] A study using 2019 NHIS data showed that these Washington Group measures classify depression and anxiety comparably to the PHQ-8 and GAD-7 respectively.[3]

© 2024 Naik H et al. *JAMA Network Open.*

eFigure: Detailed participant flow diagram

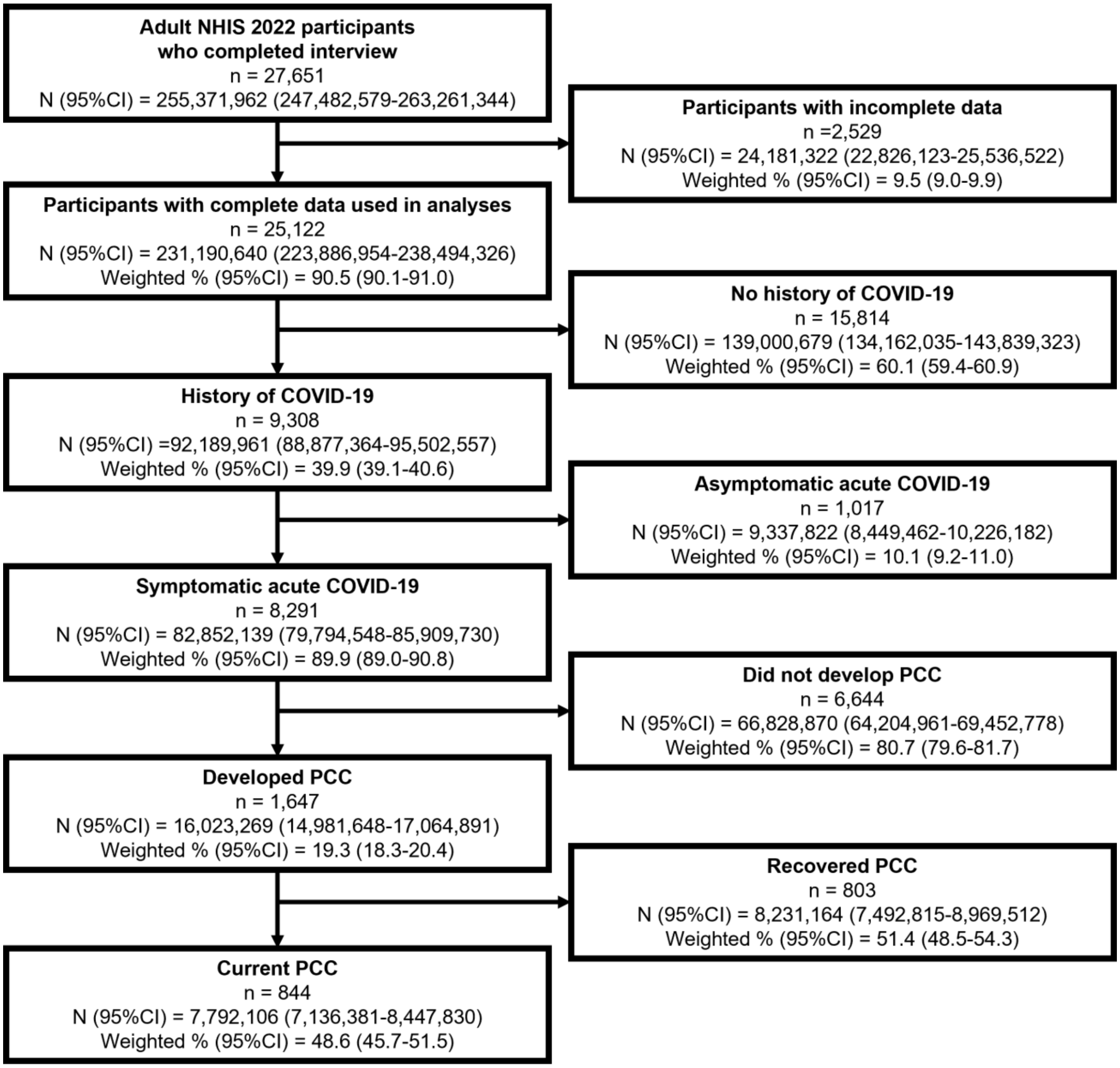

Legend: This is a more detailed version of the participant flow diagram in the **Figure** of the main text.

© 2024 Naik H et al. *JAMA Network Open.*

eTable 1: Bivariate and full multivariable models for psychiatric symptoms

	At least moderate depression symptoms (PHQ-8≥10)		At least moderate anxiety symptoms (GAD-7≥10)	
	**OR (95% CI)**	**aOR (95% CI)**	**OR (95% CI)**	**aOR (95% CI)**
**PCC Status**				
Other US Adults	1.00 [Reference]	1.00 [Reference]	1.00 [Reference]	1.00 [Reference]
Current PCC	2.64 (2.11-3.30)	1.96 (1.51-2.55)	3.17 (2.30-4.37)	2.21 (1.71-2.86)
**Interview Month (2022)**				
January-March	1.00 [Reference]	1.00 [Reference]	1.00 [Reference]	1.00 [Reference]
April-June	0.92 (0.77-1.10)	0.93 (0.77-1.13)	0.91 (0.76-1.08)	0.93 (0.77-1.13)
July-September	0.94 (0.79-1.12)	0.96 (0.80-1.16)	0.96 (0.80-1.14)	0.98 (0.81-1.17)
October-December	1.00 (0.84-1.19)	1.01 (0.84-1.22)	0.93 (0.77-1.11)	0.93 (0.77-1.12)
**Sex**				
Male	1.00 [Reference]	1.00 [Reference]	1.00 [Reference]	1.00 [Reference]
Female	1.63 (1.44-1.84)	1.52 (1.33-1.73)	1.73 (1.51-1.99)	1.65 (1.42-1.90)
**Age category, years**				
18-34	1.00 [Reference]	1.00 [Reference]	1.00 [Reference]	1.00 [Reference]
35-49	0.68 (0.58-0.80)	0.50 (0.43-0.60)	0.68 (0.58-0.80)	0.51 (0.43-0.61)
50-64	0.82 (0.70-0.95)	0.35 (0.29-0.42)	0.65 (0.55-0.77)	0.29 (0.24-0.36)
≥65	0.60 (0.51-0.70)	0.13 (0.11-0.17)	0.35 (0.29-0.42)	0.09 (0.07-0.11)
**US Region**				
Northeast	1.00 [Reference]	1.00 [Reference]	1.00 [Reference]	1.00 [Reference]
Midwest	1.08 (0.85-1.37)	1.01 (0.79-1.29)	1.02 (0.81-1.29)	0.93 (0.74-1.17)
South	1.20 (0.97-1.47)	1.09 (0.88-1.35)	1.01 (0.82-1.24)	0.91 (0.74-1.12)
West	1.28 (1.02-1.60)	1.44 (1.13-1.82)	1.01 (0.80-1.27)	1.08 (0.86-1.37)
**Urban-rural classification**				
Large central metropolitan	1.00 [Reference]	1.00 [Reference]	1.00 [Reference]	1.00 [Reference]
Large fringe metropolitan	1.05 (0.88-1.25)	1.07 (0.87-1.30)	1.09 (0.91-1.32)	1.06 (0.86-1.30)
Medium and small metropolitan	1.16 (0.98-1.38)	0.99 (0.83-1.19)	1.24 (1.04-1.48)	1.06 (0.89-1.27)
Nonmetropolitan	1.27 (1.05-1.53)	0.92 (0.75-1.12)	1.32 (1.09-1.60)	0.97 (0.78-1.21)
**Race and ethnicity**				
Non-Hispanic White	1.00 [Reference]	1.00 [Reference]	1.00 [Reference]	1.00 [Reference]
Non-Hispanic Black	1.03 (0.86-1.24)	0.79 (0.65-0.97)	0.96 (0.79-1.17)	0.76 (0.60-0.96)
Hispanic	0.92 (0.78-1.10)	0.75 (0.61-0.93)	0.85 (0.70-1.02)	0.70 (0.57-0.86)
Asian and other	0.66 (0.51-0.85)	0.59 (0.45-0.76)	0.61 (0.46-0.79)	0.53 (0.41-0.69)
**Education level**				
HS, GED or less	1.00 [Reference]	1.00 [Reference]	1.00 [Reference]	1.00 [Reference]
More than HS or GED	0.70 (0.62-0.79)	0.97 (0.84-1.12)	0.76 (0.68-0.86)	0.98 (0.85-1.14)
**Marital/ partner status**				
Not married or with partner	1.00 [Reference]	1.00 [Reference]	1.00 [Reference]	1.00 [Reference]
Married or with partner	0.51 (0.46-0.57)	0.69 (0.60-0.78)	0.61 (0.54-0.69)	0.84 (0.73-0.97)
**Employment status**				
Not employed	1.00 [Reference]	1.00 [Reference]	1.00 [Reference]	1.00 [Reference]
Employed	1.99 (1.77-2.24)	0.67 (0.57-0.78)	0.61 (0.54-0.69)	0.64 (0.55-0.76)
**Income**				
<200% FPL	1.00 [Reference]	1.00 [Reference]	1.00 [Reference]	1.00 [Reference]
≥200% FPL	0.47 (0.42-0.53)	0.77 (0.66-0.90)	0.51 (0.45-0.58)	0.78 (0.67-0.91)

© 2024 Naik H et al. *JAMA Network Open.*

eTable 1 (continued): Bivariate and full multivariable models for psychiatric symptoms

	At least moderate depression symptoms (PHQ-8≥10)		At least moderate anxiety symptoms (PHQ-8≥10)	
	**OR (95% CI)**	**aOR (95% CI)**	**OR (95% CI)**	**aOR (95% CI)**
**Health insurance**				
Not insured	1.00 [Reference]	1.00 [Reference]	1.00 [Reference]	1.00 [Reference]
Insured	1.06 (0.85-1.31)	1.01 (0.79-1.29)	1.09 (0.87-1.36)	1.14 (0.89-1.46)
**Smoking status**				
Not current smoker	1.00 [Reference]	1.00 [Reference]	1.00 [Reference]	1.00 [Reference]
Current smoker	2.17 (1.89-2.51)	1.75 (1.48-2.06)	2.28 (1.96-2.66)	1.88 (1.58-2.23)
**Functional disability**				
Not functionally disabled	1.00 [Reference]	1.00 [Reference]	1.00 [Reference]	1.00 [Reference]
Functionally disabled	8.04 (7.10-9.11)	6.44 (5.56-7.46)	5.63 (4.90-6.47)	5.10 (4.30-6.04)
**No. of medical comorbidities**				
0	1.00 [Reference]	1.00 [Reference]	1.00 [Reference]	1.00 [Reference]
1-2	1.77 (1.50-2.10)	2.03 (1.69-2.44)	1.47 (1.26-1.71)	1.72 (1.46-2.02)
3 or more	3.27 (2.75-3.89)	4.01 (3.25-4.96)	2.09 (1.76-2.49)	2.94 (2.39-3.63)
**COVID-19 vaccinations**				
0	1.00 [Reference]	1.00 [Reference]	1.00 [Reference]	1.00 [Reference]
1	1.50 (1.14-1.97)	1.77 (1.31-2.38)	1.55 (1.16-2.07)	1.84 (1.34-2.51)
≥2	0.92 (0.80-1.06)	1.28 (1.09-1.50)	0.84 (0.72-0.98)	1.25 (1.04-1.49)

Legend: This table reports the complete results for each covariate of the bivariate and multivariable logistic regressions reported in **Table 2** in the main text. Abbreviations: aOR- adjusted odds ratio; CI- confidence interval; GAD-7- generalized anxiety disorder 7-item; PCC- post-covid-19 condition; PHQ-8- patient health questionnaire-8; OR- odds ratio.

© 2024 Naik H et al. *JAMA Network Open.*

eTable 2: Associations between current PCC and psychiatric symptoms using a 5% sample of the control group (sensitivity analyses)

Outcome	Current PCC Weighted % (95% CI)	Other US Adults Weighted % (95% CI)	Bivariate	Multivariable	
			OR (95% CI)	aOR (95% CI)	*P*
At least moderate depression symptoms (PHQ-8 ≥10)	16.8 (13.8-19.7)	7.2 (5.2-9.2)	2.58 (1.79-3.73)	2.02 (1.35-3.03)	<0.001
At least moderate anxiety symptoms (GAD-7 ≥10)	16.7 (13.5-19.8)	6.3 (4.3-8.4)	2.96 (1.95-4.48)	2.56 (1.66-3.94)	<0.001
Sleep difficulties	41.5 (37.8-45.2)	24.7 (21.7-27.7)	2.16 (1.73-2.69)	1.78 (1.40-2.25)	<0.001
Cognitive difficulties	35.0 (31.0-39.0)	19.6 (17.0-22.2)	2.21 (1.76-2.78)	1.90 (1.48-2.49)	<0.001
Disabling fatigue	4.0 (2.5-5.4)	2.0 (0.9-3.0)	2.06 (1.06-4.00)	1.68 (0.91-3.12)	0.10

Legend: This table reports the results of multivariable analyses that are similar to those reported in **Table 2** and **eTable 1,** except with only a random 5% sample of the much larger control group (other US adults, N=1,153) to confirm the robustness of the main study results. All multivariable models are adjusted for interview month, US region, sex, age, urban-rural classification, race and ethnicity, education level, marital/partner status, employment status, income relative to the federal poverty line (FPL), current smoking status, health insurance, functional disability, number of medical comorbidities, and number of COVID-19 vaccinations. Abbreviations: aOR- adjusted odds ratio; CI- confidence interval; GAD-7- generalized anxiety disorder 7-item; PCC- post-covid-19 condition; PHQ-8- patient health questionnaire-8; OR- odds ratio.

© 2024 Naik H et al. *JAMA Network Open.*

eTable 3: Associations between current PCC and symptoms of depression and anxiety using different severity thresholds (sensitivity analyses)

	Current PCC Weighted % (95% CI)	Other US Adults Weighted % (95% CI)	Bivariate	Multivariable	
			OR (95% CI)	aOR (95% CI)	*P*
**Depression symptoms**					
At least mild (PHQ-8 ≥5)	40.0 (36.3-43.7)	20.7 (20.1-21.4)	2.55 (2.18-2.99)	2.11 (1.78-2.52)	<0.001
At least moderate (PHQ-8 ≥10)	16.8 (13.8-19.7)	7.1 (6.6-7.5)	2.64 (2.11-3.30)	1.96 (1.51-2.55)	<0.001
Severe (PHQ-8 ≥15)	8.0 (5.7-10.3)	2.7 (2.4-2.9)	3.17 (2.30-4.37)	2.21 (1.53-3.19)	<0.001
**Anxiety symptoms**					
At least mild (GAD-7 ≥5)	35.2 (31.4-39.0)	17.7 (17.1-18.3)	2.53 (2.13-3.00)	2.06 (1.71-2.49)	<0.001
At least moderate (GAD-7 ≥10)	16.7 (13.5-19.8)	6.3 (5.9-6.8)	3.17 (2.30-4.37)	2.21 (1.53-3.19)	<0.001
Severe (GAD-7 ≥15)	8.2 (5.9-10.4)	2.6 (2.3-2.8)	3.35 (2.45-4.57)	2.36 (1.68-3.32)	<0.001

Legend: This table reports the results of multivariable analyses for depression and anxiety symptoms that are similar to those reported in **Table 2** of the main text but use additional severity thresholds for the symptoms (“at least mild” and “severe”). Results based on the “at least moderate” threshold are included again for comparison. All multivariable models are adjusted for interview month, US region, sex, age, urban-rural classification, race and ethnicity, education level, marital/ partner status, employment status, income relative to the federal poverty line (FPL), current smoking status, health insurance, functional disability, number of medical comorbidities, and number of COVID-19 vaccinations. The main finding is that there is a similar effect estimate (odds ratio approximately 2) regardless of symptom severity. Abbreviations: aOR- adjusted odds ratio; CI- confidence interval; GAD-7- generalized anxiety disorder 7-item; PCC- post-covid-19 condition; PHQ-8- patient health questionnaire-8; OR- odds ratio.

© 2024 Naik H et al. *JAMA Network Open.*

eTable 4: Association between current PCC and psychiatric symptoms measured using WG-DEP and WG-ANX (sensitivity analyses)

Outcome	Current PCC Weighted % (95% CI)	Other US Adults Weighted % (95% CI)	Bivariate	Multivariable	
			OR (95% CI)	aOR (95% CI)	*P*
**Depression symptoms (WG-DEP)**	18.4 (15.0-21.7)	8.2 (7.7-8.7)	2.52 (2.00-3.17)	2.05 (1.59-2.64)	<0.001
**Anxiety symptoms (WG-ANX)**	39.9 (35.7-44.0)	22.1 (21.4-22.9)	2.33 (1.95-2.78)	1.89 (1.55-2.30)	<0.001

Legend: This table reports results of regression analyses for depression and anxiety symptoms that are similar to those reported in **Table 2** of the main text but use WG-DEP and WG-ANX questions for assessment of depression and anxiety respectively as opposed to PHQ-8 and GAD-7. As described in the **eMethods,** WG-DEP and WG-ANX differ in that they do not specify a timeframe for symptoms (whereas PHQ-8 and GAD-7 ask about symptoms in the prior 2 weeks). All multivariable models are adjusted for interview month, US region, sex, age, urban-rural classification, race and ethnicity, education level, marital/ partner status, employment status, income relative to the federal poverty line (FPL), current smoking status, health insurance, functional disability, number of medical comorbidities, and number of COVID-19 vaccinations. The main finding is that there is a similar effect estimate (adjusted odds ratio approximately 2 for both symptoms) regardless of the instrument measurement used to measure depression and anxiety symptoms. Abbreviations: aOR- adjusted odds ratio; CI- confidence interval; GAD-7- generalized anxiety disorder 7-item; PCC- post-covid-19 condition; PHQ-8- patient health questionnaire-8; OR- odds ratio.

© 2024 Naik H et al. *JAMA Network Open.*

eTable 5: Psychiatric symptoms in participants with current PCC compared to other COVID-19 survivors (sensitivity analyses)

Outcome	Current PCC Weighted % (95% CI)	Other COVID-19 Survivors Weighted % (95% CI)	Bivariate	Multivariable	
			OR (95% CI)	aOR (95% CI)	*P*
At least moderate depression symptoms (PHQ-8 ≥10)	16.8 (13.8-19.7)	7.2 (6.5-7.9)	2.59 (2.04-3.29)	1.91 (1.44-2.54)	<0.001
At least moderate anxiety symptoms (GAD-7 ≥10)	16.7 (13.5-19.8)	6.6 (5.9-7.3)	2.84 (2.22-3.63)	2.19 (1.65-2.91)	<0.001
Sleep difficulties	41.5 (37.8-45.2)	22.3 (21.2-23.3)	2.47 (2.10-2.92)	1.96 (1.64-2.34)	<0.001
Cognitive difficulties	35.0 (31.0-39.0)	17.5 (16.5-18.5)	2.54 (2.11-3.06)	2.09 (1.69-2.58)	<0.001
Disabling fatigue	4.0 (2.5-5.4)	1.4 (1.1-1.6)	2.98 (1.92-4.62)	2.20 (1.38-3.49)	<0.001

Legend: This table reports the results of regression analyses for depression and anxiety symptoms that is similar to those reported in **Table 2** of the main text but use other COVID-19 survivors without current PCC as the comparison group as opposed to all US adults. All multivariable models are adjusted for interview month, US region, sex, age, urban-rural classification, race and ethnicity, education level, marital/ partner status, employment status, income relative to the federal poverty line (FPL), current smoking status, health insurance, functional disability, number of medical comorbidities, and number of COVID-19 vaccinations. The main finding is that there is a similar effect estimate (adjusted odds ratio approximately 2 for all symptoms) regardless of whether participants with current PCC are compared to other US adults or other COVID-19 survivors. This suggests that the effect is specific to PCC rather than COVID-19 in general. Abbreviations: aOR- adjusted odds ratio; CI- confidence interval; GAD-7- generalized anxiety disorder 7-item; PCC- post-covid-19 condition; PHQ-8- patient health questionnaire-8; OR- odds ratio.

© 2024 Naik H et al. *JAMA Network Open.*

eTable 6: Bivariate and full multivariable models for mental health treatment uptake and cost-related barriers among participants with depression or anxiety symptoms

	No uptake of mental health treatment in past 12 months		Cost-related barrier to counseling or therapy in past 12 months	
	**OR (95% CI)**	**aOR (95% CI)**	**OR (95% CI)**	**aOR (95% CI)**
**PCC Status**				
Other US Adults	1.00 [Reference]	1.00 [Reference]	1.00 [Reference]	1.00 [Reference]
Current PCC	0.80 (0.54-1.18)	1.02 (0.66-1.57)	1.96 (1.37-2.79)	2.05 (1.40-2.98)
**Interview Month (2022)**				
January-March	1.00 [Reference]	1.00 [Reference]	1.00 [Reference]	1.00 [Reference]
April-June	1.00 (0.75-1.32)	0.81 (0.60-1.09)	0.98 (0.72-1.32)	0.97 (0.70-1.33)
July-September	0.87 (0.65-1.16)	0.79 (0.58-1.09)	1.05 (0.77-1.41)	1.03 (0.75-1.44)
October-December	0.87 (0.66-1.15)	0.81 (0.60-1.09)	1.15 (0.86-1.53)	1.04 (0.75-1.45)
**Sex**				
Male	1.00 [Reference]	1.00 [Reference]	1.00 [Reference]	1.00 [Reference]
Female	0.60 (0.50-0.74)	0.64 (0.52-0.79)	1.21 (0.96-1.53)	1.21 (0.93-1.58)
**Age category, years**				
18-34	1.00 [Reference]	1.00 [Reference]	1.00 [Reference]	1.00 [Reference]
35-49	0.79 (0.60-1.03)	1.00 (0.75-1.33)	0.76 (0.58-1.01)	0.79 (0.59-1.06)
50-64	0.81 (0.63-1.06)	1.65 (1.18-2.30)	0.40 (0.30-0.53)	0.45 (0.32-0.63)
≥65	1.00 (0.75-1.32)	3.70 (2.44-5.60)	0.16 (0.11-0.24)	0.20 (0.13-0.33)
**US Region**				
Northeast	1.00 [Reference]	1.00 [Reference]	1.00 [Reference]	1.00 [Reference]
Midwest	1.28 (0.89-1.84)	1.40 (0.95-2.06)	1.26 (0.82-1.94)	1.22 (0.77-1.92)
South	1.27 (0.91-1.77)	1.17 (0.81-1.69)	1.57 (1.04-2.38)	1.70 (1.09-2.65)
West	1.17 (0.83-1.65)	1.16 (0.78-1.70)	1.76 (1.14-2.70)	1.71 (1.10-2.66)
**Urban-rural classification[b]**				
Large central metropolitan	1.00 [Reference]	1.00 [Reference]	1.00 [Reference]	1.00 [Reference]
Large fringe metropolitan	0.78 (0.59-1.04)	0.90 (0.67-1.21)	0.83 (0.61-1.12)	0.82 (0.59-1.15)
Medium and small metropolitan	0.80 (0.63-1.02)	0.80 (0.61-1.06)	0.72 (0.55-0.95)	0.74 (0.55-1.00)
Nonmetropolitan	0.67 (0.50-0.89)	0.67 (0.48-0.92)	0.55 (0.39-0.78)	0.65 (0.45-0.94)
**Race and ethnicity**				
Non-Hispanic White	1.00 [Reference]	1.00 [Reference]	1.00 [Reference]	1.00 [Reference]
Non-Hispanic Black	2.08 (1.56-2.79)	2.30 (1.67-3.16)	1.01 (0.71-1.44)	0.93 (0.62-1.37)
Hispanic	1.66 (1.29-2.15)	1.58 (1.16-2.14)	1.26 (0.92-1.73)	0.98 (0.68-1.42)
Asian and other	1.35 (0.94-1.93)	1.49 (0.97-2.30)	1.07 (0.67-1.72)	0.83 (0.50-1.37)
**Education level**				
HS, GED or less	1.00 [Reference]	1.00 [Reference]	1.00 [Reference]	1.00 [Reference]
More than HS or GED	0.70 (0.62-0.79)	0.62 (0.48-0.79)	1.82 (1.42-2.33)	1.77 (1.31-2.38)
**Marital/ partner status**				
Not married or with partner	1.00 [Reference]	1.00 [Reference]	1.00 [Reference]	1.00 [Reference]
Married or with partner	1.00 (0.83-1.22)	1.18 (0.95-1.47)	0.93 (0.75-1.17)	0.98 (0.76-1.25)
**Employment status**				
Not employed	1.00 [Reference]	1.00 [Reference]	1.00 [Reference]	1.00 [Reference]
Employed	1.76 (1.45-2.13)	2.31 (1.76-3.03)	1.88 (1.50-2.36)	1.09 (0.83-1.43)
**Income[b]**				
<200% FPL	1.00 [Reference]	1.00 [Reference]	1.00 [Reference]	1.00 [Reference]
≥200% FPL	0.90 (0.74-1.10)	0.94 (0.73-1.20)	1.25 (0.99-1.56)	1.00 (0.76-1.31)

© 2024 Naik H et al. *JAMA Network Open.*

eTable 6 (continued): Bivariate and full multivariable models for mental health treatment uptake and cost-related barriers among participants with depression or anxiety symptoms

	No uptake of mental health treatment in past 12 months		Cost-related barrier to counseling or therapy in past 12 months	
	**OR (95% CI)**	**aOR (95% CI)**	**OR (95% CI)**	**aOR (95% CI)**
**Health insurance**				
Not insured	1.00 [Reference]	1.00 [Reference]	1.00 [Reference]	1.00 [Reference]
Insured	0.32 (0.22-0.46)	0.38 (0.25-0.56)	0.29 (0.20-0.41)	0.31 (0.21-0.48)
**Smoking status**				
Not current smoker	1.00 [Reference]	1.00 [Reference]	1.00 [Reference]	1.00 [Reference]
Current smoker	1.02 (0.80-1.30)	1.01 (0.77-1.32)	0.98 (0.74-1.30)	1.13 (0.83-1.54)
**Functional disability**[c]				
Not functionally disabled	1.00 [Reference]	1.00 [Reference]	1.00 [Reference]	1.00 [Reference]
Functionally disabled	0.67 (0.54-0.83)	0.73 (0.56-0.96)	0.72 (0.56-0.92)	1.18 (0.88-1.60)
**No. of medical comorbidities**[d]				
0	1.00 [Reference]	1.00 [Reference]	1.00 [Reference]	1.00 [Reference]
1-2	0.70 (0.55-0.91)	0.69 (0.52-0.91)	0.87 (0.66-1.16)	1.08 (0.79-1.48)
3 or more	0.51 (0.40-0.67)	0.46 (0.33-0.64)	0.43 (0.32-0.58)	0.88 (0.59-1.31)
**COVID-19 vaccinations**				
0	1.00 [Reference]	1.00 [Reference]	1.00 [Reference]	1.00 [Reference]
1	0.66 (0.42-1.03)	0.56 (0.35-0.90)	1.07 (0.66-1.74)	1.19 (0.71-2.00)
≥2	0.52 (0.41-0.66)	0.47 (0.35-0.63)	1.04 (0.77-1.39)	1.18 (0.84-1.68)

Legend: This table reports the complete results for each covariate of the bivariate and multivariable logistic regressions reported in **Table 3** in the main text. Abbreviations: aOR- adjusted odds ratio; CI- confidence interval; PCC- post-covid-19 condition; OR- odds ratio.

© 2024 Naik H et al. *JAMA Network Open.*

eTable 7: Associations between current PCC, mental health treatment uptake and cost-related barriers among participants with depression or anxiety symptoms at least "a few times a year" (sensitivity analyses)

Outcome	Current PCC Weighted % (95% CI)	Other US Adults Weighted % (95% CI)	Bivariate	Multivariable	
			OR (95% CI)	aOR (95% CI)	*P*
No uptake of mental health treatment in past 12 months	39.0 (37.1-40.9)	32.0 (25.1-38.9)	0.74 (0.54-1.03)	0.87 (0.62-1.23)	0.44
Not treated with medication	40.7 (33.3-48.1)	47.7 (45.7-49.6)	0.80 (0.58-1.10)	0.89 (0.63-1.22)	0.45
Not treated with counseling or therapy	50.9 (43.6-58.1)	63.0 (61.2-64.9)	0.63 (0.47-0.84)	0.68 (0.49-0.93)	0.02
Cost-related barrier to mental health counseling or therapy in past 12 months	30.7 (23.9-37.5)	19.3 (17.7-20.8)	1.92 (1.40-2.65)	1.88 (1.34-2.63)	<0.001
Delayed treatment	27.3 (21.0-33.5)	16.8 (15.3-18.3)	1.98 (1.44-2.71)	1.95 (1.39-2.73)	<0.001
Needed but did not receive treatment	26.7 (20.4-33.1)	16.3 (14.9-17.8)	1.91 (1.37-2.65)	1.80 (1.27-2.55)	0.001

Legend This table reports the results of regression analyses for mental health treatment uptake and barriers similar to those reported in **Table 3** of the main text, but broadens the included participants (n=4,120) to those who indicated that they had depression or anxiety symptoms at least "a few times a year" (from responses to corresponding WG-DEP and WG-ANX questions), as opposed to at least moderate symptoms in the prior 2 weeks (as captured from PHQ-8 and GAD-7). This allowed the inclusion of participants who may have had symptoms in the past but had since improved as a result of treatment. All multivariable models are adjusted for interview month, US region, sex, age, urban-rural classification, race and ethnicity, education level, marital/ partner status, employment status, income relative to the federal poverty line (FPL), current smoking status, health insurance, functional disability, number of medical comorbidities, and number of COVID-19 vaccinations. The main finding is that treatment uptake was similar between those with current PCC and other US adults, but that those with current PCC were more likely to report barriers to mental health counseling or therapy. This is similar to what was observed in the main analyses and suggests that the barriers were more likely present in current PCC, even for participants who were no longer symptomatic. Abbreviations: aOR- adjusted odds ratio; CI- confidence interval; PCC- post-covid-19 condition; OR- odds ratio.

© 2024 Naik H et al. *JAMA Network Open.*

eTable 8: Association between current PCC and mental health treatment uptake (restricted to current medications only) among participants with depression or anxiety symptoms (sensitivity analyses)

Outcome	Current PCC Weighted % (95% CI)	Other US Adults Weighted % (95% CI)	Bivariate	Multivariable	
			OR (95% CI)	aOR (95% CI)	*P*
Not on medications currently for depression or anxiety symptoms	39.8 (31.3-48.3)	50.4 (47.8-52.9)	0.76 (0.53-1.09)	0.88 (0.58-1.32)	0.53

Legend This table reports the results of regression analyses for mental health treatment uptake similar to thoe reported in **Table 3** of the main text but restricts the definition of treatment uptake to participants *currently* on medications for depression or anxiety (as opposed to medications or therapy received in the past 1 year). This excluded participants who may have been treated previously but were no longer on treatment. All multivariable models are adjusted for interview month, US region, sex, age, urban-rural classification, race and ethnicity, education level, marital/ partner status, employment status, income relative to the federal poverty line (FPL), current smoking status, health insurance, functional disability, number of medical comorbidities, and number of COVID-19 vaccinations. The main finding is that treatment uptake did not differ between those with current PCC and other adults, which is what was also observed when the broader treatment definition was used. Abbreviations: aOR- adjusted odds ratio; CI- confidence interval; PCC- post-covid-19 condition; OR- odds ratio.

© 2024 Naik H et al. *JAMA Network Open.*

eReferences

1. Adjaye-Gbewonyo D, Vahratian A, Perrine CG, Bertolli J. Long COVID in adults: United States, 2022. NCHS Data Brief, no 480. Hyattsville, MD: National Center for Health Statistics. 2023. DOI: https://dx.doi.org/10.15620/cdc:132417

2. Soriano JB, Murthy S, Marshall JC, Relan P, Diaz JV; WHO Clinical Case Definition Working Group on Post-COVID-19 Condition. A clinical case definition of post-COVID-19 condition by a Delphi consensus. Lancet Infect Dis. 2022 Apr;22(4):e102-e107. doi: 10.1016/S1473-3099(21)00703-9. Epub 2021 Dec 21.

3. Zablotsky B, Weeks JD, Terlizzi EP, Madans JH, Blumberg SJ. *Assessing Anxiety and Depression: A Comparison of National Health Interview Survey Measures*. Vol 172.; 2022. https://www.cdc.gov/nchs/products/index.htm.

4. National Center for Health Statistics. *National Health Interview Survey, 2022 Survey Description*.; 2023. Accessed September 26, 2023. https://ftp.cdc.gov/pub/Health_Statistics/NCHS/Dataset_Documentation/NHIS/2022/srvydesc-508.pdf

© 2024 Naik H et al. *JAMA Network Open.*
