# Supplementary material for: Psychiatric Symptoms, Treatment Uptake, and Barriers to Mental Health Care Among US Adults With Post–COVID-19 Condition
Source: JAMA Netw Open. 2024 Apr 25;7(4):e248481. doi: 10.1001/jamanetworkopen.2024.8481 (PMC11046346; doi:10.1001/jamanetworkopen.2024.8481)
Supplement: Supplement 2. — Data Sharing Statement [file jamanetwopen-e248481-s002.pdf]

Data Sharing Statement

Naik. Psychiatric Symptoms, Treatment Uptake, and Barriers to Mental Health Care Among US Adults With Post–COVID-19 Condition. *JAMA Netw Open*. Published April 25, 2024. doi:10.1001/jamanetworkopen.2024.8481

Data
Data available: Yes
Data types: Deidentified participant data
How to access data: https://www.cdc.gov/nchs/nhis/2022nhis.htm
When available: With publication

Supporting Documents
Document types: Statistical/analytic code
How to access documents: SAS code pertaining to analyses will be available from the authors upon reasonable request.
When available: With publication

Additional Information
Who can access the data: To anyone with a reasonable request.
Types of analyses: For the purpose of research studies.
Mechanisms of data availability: With investigator support.
Any additional restrictions: None
